# Using Experimental Research Designs to Explore the Scope of Cumulative Culture in Humans and Other Animals

**DOI:** 10.1111/tops.12391

**Published:** 2018-10-30

**Authors:** Christine A. Caldwell

**Affiliations:** ^1^ Division of Psychology University of Stirling

**Keywords:** Cumulative culture, Cultural evolution, Comparative psychology, Transmission chain, Ratchet effect, Human, Nonhuman, Experimental design

## Abstract

In humans, cultural evolutionary processes are capable of shaping our cognition, because the conceptual tools we learn from others enable mental feats which otherwise would be beyond our capabilities. This is possible because human culture supports the intergenerational accumulation of skills and knowledge, such that later generations can benefit from the experience and exploration efforts of their predecessors. However, it remains unclear how exactly human social transmission supports the accumulation of advantageous traits, and why we see little evidence of this in the natural behavior of other species. Thus, it is difficult to know whether the cognitive abilities of other animals might be similarly scaffolded by processes of cultural evolution. In this article, I discuss how experimental studies of cultural evolution have contributed to our understanding of human cumulative culture, as well as some of the limitations of these approaches. I also discuss how similar research designs can be used to evaluate the potential for cumulative culture in other species. Such research may be able to clarify what distinguishes human cumulative culture from related phenomena in nonhumans, shedding light on the issue of whether other species also have the potential to develop cognitive capacities that are outcomes of cultural evolution.

## Abstract 2

Culture drives cognitive evolution by supporting the transmission and intergenerational accumulation of skills and knowledge, based on social learning and teaching: Later generations benefit from what their predecessors acquired. Taking a metaperspective on those experimental studies that explore the mechanisms underlying cultural transmission, Caldwell discusses their potential for generating valuable insights, their possible limitations, and their generalizability to other species.

## 1 Introduction

### 1.1 The cultural evolution of cognition: A uniquely human phenomenon?

In considering the role of cultural evolution in shaping cognition, it is important to consider the scope and constraints of any such effects, including whether these are restricted to humans alone. There are now many widely accepted examples of cultural transmission in nonhumans, so does it follow that some of the cognitive capacities of these animals might also be influenced in nontrivial ways by cultural inputs? Or is human cultural evolution fundamentally different, and potentially capable of supporting the transmission of cognitive tools in ways that nonhuman cultural evolution simply cannot? In the current article, I examine how key features of human cultural evolution can be captured in experimental research designs, and I describe how such experimental methods can be used to shed light on what might distinguish human cultural evolution from similar phenomena in nonhumans.

It is now relatively uncontroversial to claim that at least some aspects of the cognition of modern humans are largely a consequence of cultural evolution, built up over generations of social transmission, rather than biological predispositions shaped primarily by genetic control. Admittedly, there are some striking differences in opinion regarding the range of attributes to which this might apply. For example, some controversy remains over the extent to which cultural evolution might account for human capacities for theory of mind (e.g., compare Heyes & Frith's, 2014, cultural evolutionary account, with claims of false belief attribution in infancy, for example, Onishi & Baillargeon, 2005). But for other examples, such as number systems and notations, algorithms for calculation, or graphical codes for record‐keeping of any kind, few would question the importance of cultural evolution as an explanation for the use of such conceptual tools. There is historical evidence documenting the development of these techniques over many generations (Grattan‐Guinness, 1997), they show cultural variability (e.g., Bender & Beller, 2014), and mastery of the skills typically requires conscious effort on the part of the learner (and indeed these may need to be explicitly taught).

However, if some of our human cognitive abilities are not a direct outcome of specialized human biology, but are instead learned (e.g., Heyes, 2018a), then this poses the important question of whether such abilities may, therefore, be possible in other species. We are by no means the only species that exhibits cultural traditions (e.g., Whiten et al., 1999), so does this learning build and bolster cognitive capabilities in these animals too, as well as influencing their (more readily observable) behavioral traits?

### 1.2 Human cultural evolution in comparative perspective

The answer to the above question depends on how similar human cultural evolution is to related processes observed in other species. However, questions remain over the precise nature of the differences between human cultural evolution and cultural transmission in other species, as well as the degree of similarity (e.g., Dean, Vale, Laland, Flynn, & Kendal, 2014). Experimental research designs are likely to prove critical in resolving these issues. Although naturalistic observations of both human and nonhuman cultural traditions can be extremely enlightening, experimental research offers important advantages. First, in humans, such designs make it possible to manipulate the conditions under which learning can occur, permitting conclusions about prerequisites and constraints associated with particular population‐level outcomes. Second, in nonhumans, it is possible to actively probe the limits of a species’ capabilities, the extent of which may not be apparent from the available naturalistic evidence. In the following sections, I consider the existing and potential contributions of both of these experimental approaches, with regard to similarities and differences between human and nonhuman cultural processes.

## 2 Laboratory studies of cultural evolution in human participants

### 2.1 Operationalizing human cultural evolution in experimental research designs

#### 2.1.1 Capturing significant aspects of cultural evolutionary change

As noted above, experimental research offers potential for manipulation of variables of interest in order to determine critical prerequisites for particular human cultural processes. This is a key step in understanding whether certain cultural processes might also be present in other animals, since it is possible to establish whether the effects are underpinned by mechanisms that are unique to humans (e.g., language) or shared with other species. However, to do this one must first address the issue of precisely which features of human culture we wish to capture experimentally, and how to go about operationalizing these features for measurement. The phenomenon of *cumulative culture*, or *cumulative cultural evolution*, has frequently been cited as exemplifying what is particularly special about human culture (Boyd & Richerson, 1996; Heyes, 2012; Hill, Barton, & Hurtado, 2009; Laland & Hoppitt, 2003; Tennie, Call, & Tomasello, 2009). This is partly because it is believed to be either absent or extraordinarily rare in the natural behavior of nonhumans. However, it is primarily due to the number of other peculiarly human traits it appears to support (e.g., Tomasello, 1999). This latter point is of course particularly relevant from the perspective of the issue at hand, that is, the potential for shaping of cognitive abilities.

Cumulative cultural evolution can be conceived as a distinct subcategory of cultural evolution, distinguished by its creation of traits in later generations that would also have been preferred by members of earlier generations. In operationalizing this phenomenon for experimental research, we are, therefore, looking for cases where the traits expressed in later generations are demonstrably more effective or appealing in ways that are not purely dependent on current conditions. This criterion helps to distinguish cumulative culture from otherwise potentially confusable cases in which cultural evolution generates change that is cumulative only in the general sense of being incremental and historically dependent, but without producing traits that are increasingly advantageous. Tomasello (1990, 1999) famously applied the analogy of the ratchet to human culture as a means to capture this same notion, describing human cumulative culture as exhibiting a “ratchet effect.” This apt analogy highlighted this particularly interesting and powerful property of cultural evolution, differentiating it from change that is more arbitrary and/or cyclical.

It should be noted that the notion of contextually independent trait value may be considerably more problematic for some behaviors compared with others. Communicative signals, for example, are only effective if they can be interpreted by receivers. The effectiveness of signal form is, therefore, necessarily tied to context. As such, the specifics of communicative conventions are likely to be subject to change that is less ratchet like. Nonetheless, increases in expressive power, independent of the nature of the signals used (e.g., allowing for communication of novel concepts, or more efficient communication of existing ones), remains understandable as cumulative in the sense of the cultural ratchet.

Therefore, in attempting to capture the phenomenon of cumulative culture under laboratory conditions, a key feature which needs to be reflected is the potential to deliver tangible benefits to learners in later generations. Other features of cumulative culture which have been identified in the existing literature have tended to arise from descriptions of exemplar cases, rather than specifications of minimal defining criteria. But if our motivating interests are, first, identifying significant critical preconditions, and second, establishing the extent of similar effects in other species, then it is important to focus on features we consider to be core constructs, as opposed to attempting to simulate phenomena which correspond closely to portrayals of the most striking examples of human cumulative culture.

To illustrate this point, consider the nature of the changes proposed to arise from cumulative culture. In some of the literature, cumulative culture has been characterized as being best illustrated by increases in the complexity of cultural traits (Dean et al., 2014), with increases in efficiency sometimes being cited alongside, as another possible outcome that is somehow inferior or less interesting (see Section V.(2) in Dean et al., 2014). In contrast, other authors have emphasized the advantages associated with increasing efficiency and compressibility, with outcomes relating to complexity proposed to be less predictable (Kirby, Tamariz, Cornish, & Smith, 2015).

However, conceptualizing cumulative culture more broadly as allowing the development of increasingly preferred traits over learner generations helps to clarify the apparent dissent over the expected outcomes of cumulative culture. In some instances, increased complexity is associated with increased functionality (e.g., modular technological innovations), whereas in others (such as refinements of existing technologies) efficiency is desirable. Therefore, although cumulative culture often can be associated with increasing complexity, this will not necessarily be the case. Likewise, it can be associated with increasing efficiency, but this is also by no means guaranteed. Indeed, other benefits may be associated with cumulative cultural traits which are not easily captured as either complexities or efficiencies. For example, Schofield, McGrew, Takahashi, and Hirata (2018) proposed adding “security” and “convenience” to this list, along with complexity and efficiency. Talking of “preferred” traits may seem altogether more vague and difficult to evaluate objectively, but it does at least allow us to capture what it is about cumulative culture that we believe to be so valuable and compelling, that is, its capacity to deliver advantageous traits, without placing restrictions on the nature of those advantages (they would not, for example, need to be associated with demonstrable fitness benefits). This inclusive concept of preference also effectively encompasses many noteworthy aspects of human culture, which most would agree is broad in scope, extending over a range of diverse domains including useful technologies, highly organized rule‐governed societies, and suites of technical knowledge and skills that permit survival in otherwise inhospitable natural habitats.

In practice, it might turn out to be difficult to distinguish traits that would be preferred over their precursors in an absolute sense, from those which are favored only due to current environmental conditions. Nonetheless, regardless of the difficulties of evaluating particular real‐world cultural traits according to this criterion, it is relatively straightforward to implement within the context of experimental research with human participants, since explicit task goals can be specified against which performance can be objectively evaluated, making quantifiably better solutions easy to identify. For examples, see the next section, describing studies using flight distances of paper planes and heights of spaghetti towers (Caldwell & Millen, 2008), measures of similarity to a target goal state (Muthukrishna, Shulman, Vasilescu, & Henrich, 2014), and load mass of baskets (Zwirner & Thornton, 2015).

#### 2.1.2 Capturing the trans‐generational cumulative potential of social learning

Experimental designs aiming to capture cumulative culture must also effectively capture the trans‐generational nature of the accumulated learning benefits. Simply demonstrating that social information can be beneficial is not quite sufficient to conclude that there is potential for cumulative culture. It is important to be able to show that the benefits of social learning stack up over multiple generations of transmission.

Experimental designs intended to capture cumulative culture must, therefore, incorporate multiple learner generations. However, taking into consideration the ultimate goal of drawing comparisons with nonhuman populations, we are once again faced with the question of specifying minimum criteria. Assuming that the first learner generation of any experimental design is comprised of individuals who approach a task or problem in the absence of any social information (thus providing a baseline of the likely success of naïve task exploration), over how many subsequent generations must any such measures of success be seen to accumulate?

In order to conclude that the experimental results suggest potential for cumulative culture, transmission to only one generation of social learners (exposed to information from the asocial learners’ baseline attempts) would seem insufficient. It is difficult to compare the relative costs of learning from social demonstrations versus individual exploration, and this limits what can be concluded from comparing performances of participants who complete a task using individual exploration alone, with those of participants who have an opportunity to learn from such baseline exploration attempts before making their own. In this scenario, even if the social learners performed better, it is not necessarily possible to conclude that this could equate to a benefit to later learner generations in the real world, since finding an appropriate source of social information, or the time consumed observing them, could easily be at least as costly as simply expending additional individual exploration effort to reach an equivalent level of performance. However, if it is possible to show that comparable social learning opportunities are more valuable in later generations, then it is much more reasonable to conclude that later generation learners are liable to be in a more advantageous position regarding benefits of social information, compared with their predecessors. So, for example, if a further sample of participants were given the opportunity to learn from individuals who had observed the attempts of the asocial learners, and if this second generation of social learners outperformed their socially learning predecessors, then it is possible to conclude that the social information itself was increasingly valuable.

If this is the case, then we can make the argument that benefits can, in principle, accrue with repeated transmission. Such a demonstration, therefore, identifies the potential for cumulative culture, regardless of whether it results in an outcome that is identifiably beyond what a single individual could achieve in principle by themselves. Thus, although cumulative culture is sometimes described as resulting in “behaviours that no individual could invent on their own” (Boyd & Richerson, 1996, p. 770), as Tennie, Caldwell, and Dean (2018) have previously argued, this should not be used as a criterion for guiding classification of ambiguous cases, as it will tend to rule out examples which represent incipient cumulative culture. Also, and more pragmatically, from the perspective of experimental research design, feasibility issues dictate that measured behaviors must be relatively achievable within a contracted timeframe. Thus, it may be more helpful to think of the core defining feature of cumulative culture in terms of the potential for increasingly valuable shortcuts to learning available to social learners over generations of transmission.

The studies reported in Caldwell and Millen (2008) provide examples of how benefits of experience can be shown to accumulate over learner generations in an experimental context. Successive learner “generations” of participants (who could observe and interact with their immediate predecessors) were each required to complete the same task, all scored according to a prespecified goal measure. All participants were given the same time limit in which to complete the task, as well as the same materials. Furthermore, they each had the same amount of time available for observing their predecessors. The time periods were short (5 min of observation time, followed by 5 min of building time) so the tasks were necessarily relatively simple. In one experiment, participants were required to build a paper plane (the goal measure being flight distance), and in another, the task was to build a tower from raw spaghetti and modeling clay (the goal measure being tower height). In both cases, participants in later generations scored higher in relation to these goal measures compared with those in earlier generations. Therefore, these participants were able to achieve better outcomes in an equivalent time period, including the time available for observational learning. Presumably, the performances to which they were exposed were valuable sources of information about how to maximize their own performance, and importantly, more so than the performances of members of earlier generations would have been.

Similar research designs have since been used by other authors to demonstrate similar effects of accumulating benefits of otherwise equivalent social learning opportunities. For example, Muthukrishna et al. (2014) asked participants to try to match a target image using an unfamiliar image editing software package. In one of their experimental conditions (see Experiment 1, five‐model condition), they found that those in later generations produced images that were quantifiably more similar to the target. Zwirner and Thornton (2015) presented participants with a basket‐making task, providing them with everyday materials such as paper, string, and rubber bands, and a goal of producing a basket capable of transporting rice. They found that participants in later generations were able to perform better according to the goal measure of the mass of rice successfully transported.

In these studies, it has, therefore, been possible to show that the benefits of social learning could accrue with repeated transmission, such that learning from individuals who had themselves been exposed to social information was typically even more valuable than learning from individuals who had engaged in naïve exploration. These laboratory models capture this powerful property of human cultural transmission, which allows social learners to benefit not just from the experience of others, but the accumulated experience of multiple others, even when opportunities for learning are otherwise held constant.

It should perhaps be noted at this point that the benefits of experience can accumulate over multiple generations even when the “social” information is experienced in a decidedly nonsocial context. Muthukrishna et al. (2014) passed information between participants in the form of written instructions, so the individuals in question never actually encountered one another directly. In Zwirner and Thornton's (2015) study, learning from others’ completed attempts was found to be sufficient to generate improvements in performance. This was also the case in the follow‐up to Caldwell and Millen's (2008) study (Caldwell & Millen, 2009), described in the next section. Thus, it remains an empirical question whether or not the “social” aspect of some forms of social learning actively facilitates learning, and this does not necessarily have any bearing on the potential for the accumulation of benefits of experience.

### 2.2 Value and limitations of experimental research design for understanding prerequisites of cumulative culture

One of the benefits of developing laboratory models, such as those described, is that this allows manipulation of particular variables of interest, to compare group‐level outcomes when members complete the tasks under different conditions. From the perspective of understanding the potential scope of cumulative culture, including the extent to which such effects are likely to be possible in other species, a key question concerns the cognitive and behavioral prerequisites of this phenomenon. This question can, therefore, be addressed experimentally, through manipulation of the availability of sources of information, or constraints placed on participants’ strategies or resources.

However, it should be emphasized that results from any such simplified laboratory simulation must be treated with due caution. Most readers of this article will likely be familiar with the aphorism that “all models are wrong but some are useful,” attributed to Box (e.g., 1979), so I will not waste time elaborating on its meaning here. The important point from the perspective of the current article is that the same could equally well be said of cultural evolution experiments. While all experimental research necessitates simplification of the noise and complications of real‐world phenomena, with consequent impact on external validity, cultural evolution experiments arguably represent a particularly extreme case. To preserve their usefulness, we must be mindful of the simplifications imposed.

The need to control factors such as population size and structure render contexts inevitably unrealistic, but the most serious threats to generalizability arise from the necessary differences in scale between experimental and real‐world contexts. This applies most obviously to the timescales involved, with individual participants’ involvement in laboratory experiments typically lasting under an hour. In contrast, the behaviors that these studies are intended to help explain are generally thought to have developed over multiple human lifespans. This scaling down of timeframes places inherent constraints on the tasks that can be presented, with these correspondingly scaled down in their complexity due to the time available for completion. The reliance on simple tasks presents an obvious limitation in that it could be argued that findings from cultural evolution experiments might not extend to any behavior believed to depend on cumulative culture in the real world. In studies investigating the conditions necessary for cumulative culture in humans, these inevitable simplifications impose significant limitations on the types of conclusions that can be drawn, and the nature of the evidence that would be required to draw conclusions of any kind.

So, for example, it is important to note that failure to find an effect of cumulative improvement over multiple learner generations within laboratory transmission chains is not necessarily an indication that some missing element is, therefore, a prerequisite for cumulative culture, since there are likely to be a multitude of alternative reasons why an effect might not have been found. In contrast, finding a positive effect of improved performance over multiple generations of social transmission is more informative. This can allow a researcher to establish that some variable of interest which has been excluded is not a strict prerequisite for cumulative culture. However, the important caveat arising from the necessary simplifications and reductions of scale is that this might well be context dependent. The excluded variable of interest may, therefore, turn out to be necessary for cumulative improvements to be observed in other tasks, or within different population structures.

A further type of result might involve multiple experimental conditions across which a variable of interest had been manipulated. Finding a positive effect of cumulative improvement in one condition, and a significantly reduced effect in at least one other, allows a researcher to conclude that this manipulated variable may be important for cumulative culture. The caveat once again, however, is that this is likely to be context specific. As a consequence, any individual experiment can only ever contribute a small part of the explanation of how human minds generate cumulative culture, and why those of other species appear not to. Ultimately, this question must be approached from a variety of different angles in order to form a more complete picture.

It is perhaps helpful to illustrate these points about what can and cannot be concluded from such experimental research designs by considering Caldwell and Millen's (2009) study, which was the follow‐up to the Caldwell and Millen (2008) publication described earlier. Caldwell and Millen (2009) used the paper aeroplane task paradigm established in the earlier (2008) publication in order to compare conditions in which participants had access to different sources of social information (through observation of others’ task completion, inspection of their completed artifacts, and opportunities for exchanging information through spoken communication). The results indicated that cumulative improvement was possible on the basis of any of these sources of information. From a theoretical perspective, the most interesting conclusion was that cumulative culture appeared to be possible even in the absence of opportunities for imitation or teaching. In line with the caveats noted above, the conclusion that these are not strict prerequisites can be justified, whereas a blanket claim that they are unimportant, or unnecessary in *any* context, cannot.

### 2.3 Investigating task‐specific effects in human cultural evolution

Even though conclusions about the prerequisites for cumulative culture will be determined in part by the properties of the task in question, this should not be regarded as condemnatory for cultural evolution experiments. Far from closing doors, this perspective illuminates previously underexplored avenues of investigation, which offer potentially rich insights to a more comprehensive and nuanced understanding of the distinctiveness of human culture. Indeed, this is particularly advantageous from the perspective of establishing the potential scope of cumulative cultural effects under different conditions, including whether this might extend to more abstract contexts such as cognitive skills.

Turning the inevitable reality of task‐specific effects into an advantage, rather than a limitation, simply entails direct manipulation of task variables themselves, to investigate how these affect transmission requirements. For example, Caldwell, Renner, and Atkinson (2017) investigated the transmission of knot‐tying skills for knots of differing complexity. Participants had access to either finished products alone, or had additional pictorial instructions about the process of completion, or were also paired with another participant who had mastered the skill and could, therefore, engage in active demonstration and interactive instruction and feedback. While all transmission conditions were equally effective for the knots classified as simple, the interactive teaching condition was much more effective for those knots classified as complex.

It should be emphasized that this particular example was not a study illustrating cultural evolution, since it considered only transmission to a single generation of learners. Furthermore, since it concerned retention of an experimentally introduced complex skill, only loss can be measured, and therefore there is no way to assess the kind of improvements in performance that would be indicative of potential for cumulative culture. Naturally, such approaches are likely to generate very different conclusions about prerequisites for effective transmission, with studies of loss possibly identifying mechanisms supporting high‐fidelity copying as more important than they would be for cumulative improvement, and likely also underestimating the importance of mechanisms generating effective novel inventions and modifications. Nonetheless, the logic behind the experimental design in Caldwell et al.'s (2017) study can also be applied to cultural evolution experiments, in order to investigate the differing requirements for cumulative improvement within different behavioral contexts.

## 3 Laboratory studies of cultural evolution in nonhuman participants

Of course, studying only humans can only provide one part of the picture of the potential scope of cumulative culture. If a key question of interest concerns the extent of such effects in other species, then studies of nonhuman animals also provide a critical source of evidence. Although cumulative culture is widely believed to be either very rare or completely absent in the natural behavior of other species (as noted previously), it stands to reason that it should be possible to document some degree of continuity between ourselves and our closest evolutionary relatives, regarding the tendencies and capacities that support such effects, even if the phenomenon itself does not manifest in quite the same form.

Even within the spontaneously occurring behavior of wild populations, there are putative examples of cumulative culture from nonhumans. For example, see Schofield et al.'s (2018) analysis of historical data on Japanese macaque foraging techniques, and Sanz, Call, and Morgan's (2009) report of chimpanzees’ tool modification. However, these examples remain controversial due to the difficulty of ruling out potential alternative interpretations (such as modifications reflecting asocially learned refinements introduced as a consequence of individuals’ increasing personal experience of the particular foraging technique or tool, shaped by environmental feedback alone).

Experimental methods make it possible to systematically investigate the capabilities of other species, using designs specifically devised to elicit key evidence, allowing clearer conclusions regarding whether criteria for cumulative culture have been fulfilled. In addition, from the perspective of the question in hand (i.e., that of the potential scope of cumulative culture in nonhumans, in terms of the traits it might support), experimentation can also establish the kinds of skills that could in principle be supported by cumulative culture in a given species.

In terms of the details of the designs which can be used to establish the potential for cumulative culture in nonhumans, the same principles of design apply as already discussed for humans. If we are primarily interested in the accumulation of benefits of collective experience, then once again we should be looking for evidence of objective benefit to later generations, and social information becoming increasingly valuable.

In fact, currently there are very few studies with nonhumans that follow the principles of design set out in Section 2, although I will describe one which does. However, before doing so, it is important to note that even when using experimental designs which are in principle capable of demonstrating the accumulation of benefits of experience, we must still be wary of what we can, and cannot, conclude. The very same limitations that apply to human cultural evolution experiments (described in Section 2.2) also apply to attempts to use similar research designs with nonhumans.

So, once again there are limits on what we can conclude from a null result. If we find no evidence of later generations of learners being able to benefit from the accumulated expertise and exploration effort of their predecessors, this could occur for any one or more of a number of reasons that may not preclude potential for cumulative culture in other contexts. It might be that the performances that would be required for later generations to demonstrate improvements upon those of their predecessors are simply beyond the capabilities of that species. The individuals might never be able to learn the necessary behaviors under any circumstances. Alternatively, it could be the case that although the task can in principle be mastered to the required degree, the opportunities afforded for learning from others in the context of the research design are not adequate for expertise to be effectively transferred (e.g., reliant on learners paying close attention to a relatively brief performance by an experienced individual—who may be unfamiliar, uninteresting, or potentially antagonistic).

Therefore, just as a failure to identify trans‐generational improvements in task performance in humans does not allow us to draw firm conclusions about necessary preconditions, similar failures in studies of nonhumans likewise do not allow us to make sweeping generalizations about the potential for members of that species to accumulate benefits of experience via social transmission. Also, and again corresponding to the limitations identified in relation to studies of humans, even positive results must be treated with due caution. Positive results, while demonstrating that cumulative improvements are possible, should generally be assumed to be context specific, at least until shown to generalize across a range of varying contexts.

However, echoing the optimistic message in relation to studies involving human participants, the fact that a positive result is likely to be dependent on the details of the task, as well as other contextual aspects, should be viewed as an opportunity rather than a threat. This is in fact particularly true for studies of nonhuman species, because this may allow researchers to design experiments in which later learner generations can benefit from the accumulated experience of their predecessors, through careful consideration of the propensities of the species in question.

Sasaki and Biro's (2017) study of the efficiency of routes taken by homing pigeons represents an excellent example of such an approach. The researchers took into account the birds’ tendency to fly alongside a partner, or as part of a flock, when released together. This flocking naturally influenced choice of route, smoothing out idiosyncratic deviations. The researchers reasoned that this process could result in the birds learning more efficient routes, and that a replacement design involving multiple learner generations might, therefore, produce later generations who flew more efficient routes compared with earlier ones. Thus, the researchers took advantage of the flocking tendency, which generated social heritability of the trait in question (i.e., route choice). They also permitted the birds within any given generation (i.e., particular pairs) multiple flights together, so there were opportunities for each pair to further refine route choice based on direct experience. Thus, across 10 chains, each composed of five pigeons (each of which flew 12 times with their predecessor in the chain, then 12 times with their successor), routes flown by pairs in the final generations were more efficient than routes flown by pairs in earlier generations.

This result certainly fulfils the criterion of demonstrating tangible benefit to individuals in later generations as a consequence of the accumulated experience of members of earlier generations. It is reasonable to assume that the “goal” of the homing pigeons is to complete their journey as quickly and efficiently as possible, so it follows that the birds from earlier generations would—in principle—also have preferred the shorter, faster routes of their successors. In studies of nonhumans, establishing the unambiguous superiority of certain traits over others is likely to pose particular challenges. As previously noted in relation to experiments involving human participants, the ability to present an explicit task with clearly defined goal measures (e.g., tower height, maximum load mass, or similarity to a target) renders this requirement fairly trivial. But it is of course impossible to simply induce task‐appropriate motivations in nonhumans in quite the same way. Thus, researchers must rely upon their subjects’ preexisting natural motivations. In some cases (such as Sasaki & Biro's study) it may be possible to design an experimental paradigm which embeds this motivation within its original functional context. However, task motivation is more likely to be induced through pretraining, as a means to ensure that subjects associate aspects of experimental task performance with primary reinforcers (such as food) of varying quantity or quality.

A further important detail about Sasaki and Biro's study is that the birds had equivalent opportunities for direct personal experience of flying the routes (i.e., same number of flights completed, in the respective roles as the experienced, and inexperienced, member of a pair). This means that it was possible to compare like with like, in considering the performances of birds from different generations. Thus, we can also be confident that the later generation birds are able to profit from accumulated expertise and exploration effort even in the context of learning opportunities that were otherwise matched to those of their predecessors. The later generation birds performed better because the social information to which they were exposed was apparently more valuable.

It is important to note, however, that simplified task designs might demonstrate effects which *in practice* do not have that effect in real‐world populations. In real human cultures, we know that later generations (this time also in the “real” sense of population turnover) genuinely learn from, and build on, the accumulated expertise of their predecessors, with systems and technologies being continually refined over many lifetimes. And as such, we do routinely make use of techniques and inventions that were unavailable to our ancestors. So in that respect we know that cumulative culture is a real phenomenon in human societies. In contrast, it remains to be seen whether the effects identified in Sasaki and Biro's research, for example, illustrate effects which would actually bring tangible benefits to later (real) generations of birds, compared with their predecessors. So, do migrating species develop increasingly efficient routes over many years, in spite of complete population turnover? Do contemporary populations of migrating birds use routes which would have been used by their conspecific counterparts in decades gone by, had they only had exposure to these possibilities? Sasaki and Biro's (2017) findings cannot answer these questions, but they do hint at their plausibility.

Regardless of whether this effect reflects any real‐world intergenerational learning effects or not, it is likely that many readers will in any case find Sasaki and Biro's claims of cumulative culture questionable. The route efficiencies of homing pigeons may not represent an example of cumulative culture as most would ordinarily conceive of it. Such concerns are valid and should not be dismissed. In fact, such observations are essential for understanding how we can evaluate the extent to which nonhuman capacities for cumulative culture approach our own.

This is because task simplification, and careful tailoring of research designs to the competencies of the species in question, may well be our most effective means of approaching the question of how close other animals can come to human‐like culture. As noted previously, drawing informative conclusions depends on being able to identify positive effects, whether this might be a standalone positive result, or a result that demonstrates a significant difference between a positive effect of cumulative performance in one condition, and a reduced effect in another. However, subscribing to this view, that positive effects are far more informative than null results, could potentially result in abandonment of studies of nonhumans as an exercise in futility. If ultimately our endeavor is to evaluate claims about cumulative culture being a uniquely human trait (especially if we regard those claims to be well‐founded), then we might conclude that there is little to be gained from nonhuman experimental research if its success depends on finding positive effects.

However, this is precisely the reason why the likely existence (and indeed virtual inevitability) of task‐specific effects should be viewed not as a threat to the validity of our research conclusions, but rather as the key to progress in this field. Simplified task designs, which nonetheless preserve the integrity of the fundamental value of cumulative culture, may be our only means of escape from this logical quandary of identifying positive evidence of a property which so far has only been identified in human cultural traditions. And regardless of the specifics of the task in question, if it is possible to demonstrate that learners are able to benefit from limited exposure to others’ performance such that they can take advantage of the accumulated expertise of multiple learner generations, then we have captured an unarguably powerful property of social learning.

Such research designs can also then provide a starting point for identifying the constraints on cumulative culture‐like effects in other species. Therefore, somewhat similar to the approach taken in the study of teaching of knot‐tying described previously (Section 2.3), critical task variables can be manipulated according to researchers’ expectations about the conditions under which a cumulative culture‐like effect may be possible in their study species. Researchers may, therefore, be able to identify limiting factors which restrict the extent to which we see such effects occurring in natural behaviors.

## 4 Cumulative culture and the cultural evolution of cognition

Returning to the focus of this special issue, we must also consider the implications of the arguments presented here for our understanding of the cultural evolution of *cognition*, specifically. Following on from the point above, suggesting that it might be possible to document cumulative cultural phenomena in other species, if in very restricted set contexts, I would be inclined to speculate that such contexts are unlikely to extend to the kinds of conceptual tools that actively facilitate and shape thinking.

Cumulative culture is necessarily dependent on agents being influenced by social information in how they approach their goals. In humans, this can occur across a wide variety of contexts, due to an explicit recognition of the potential value of social information (which Heyes might describe as explicitly metacognitive social learning, for example, Heyes, 2016). In contrast, it is reasonable to assume that nonhuman animals lack such explicit understanding, given the contentiousness of claims of metacognitive awareness in nonhumans, even as these relate only to the individual's own knowledge state, (Hampton, 2009). Therefore, it is likely that there are major constraints on the contexts within which nonhumans attend and respond to social information. This is probably restricted to two broad categories of circumstance. The animal could either possess a specialized mechanism that generates behavioral heritability, shaped by natural section (similar to the flocking tendency underpinning Sasaki & Biro, 2017, findings). Alternatively, the animal's own experience could have resulted in the formation of associations such that conspecific behavior becomes a cue indicating that certain behavioral responses are likely to have positive consequences.

In either such circumstance however, although faithful social transmission can occur, it is likely to do so only in very restricted contexts. In the case of naturally selected tendencies, these will likely only operate within the particular domain which created the selective pressure for those mechanisms to exist (e.g., route choice for the pigeons). To illustrate this point, consider the fact that although it might be possible to train pigeons to have a “conversation” (Epstein, Lanza & Skinner, 1980), we would not expect naïve untrained pigeons to be able to spontaneously take over one of the roles in this performance, on the basis of simply observing their trained counterparts’ interaction. Although high‐fidelity transmission may be possible for route choices, it is not likely to operate in this novel context.

Associative learning mechanisms provide another possible source of transmission fidelity which may have the power to support cumulative culture‐like effects in nonhumans. Through experience, animals may learn to use cues from conspecifics as predictors of likely reinforcement (e.g., Leadbeater & Chittka, 2009). However, although an associatively learned tendency to copy could have the potential to generalize to a degree that permitted reproduction of novel variants (e.g., Custance, Whiten, & Bard, 1995), which would be necessary for cumulative enhancements to accrue at all, this would also be unlikely to extend much beyond the domain within which it was originally learned.

If it is true that cumulative culture‐like effects in nonhumans are restricted to cases implicating one or other (or possibly some combination) of these two routes to transmission fidelity, we are unlikely to observe these supporting the development of cognitive skill. This would require extending the reach of social learning mechanisms, developed or selected for within particular behavioral domains, into a completely novel domain involving abstract rules with opaque benefits and functions.

In contrast, if cumulative culture in humans relies heavily on explicitly metacognitive social learning (Heyes, 2018b), with learners actively seeking out relevant information based on their inferences and assumptions about others’ knowledge, this considerably broadens out the behavioral contexts for which increases in functionality could be observed over generations of social transmission, potentially opening up the possibility of the cultural evolution of more abstract cognitive functions.

## 5 Conclusions

In conclusion, experimental approaches can, therefore, contribute a great deal to understanding the potential scope of cumulative culture, including the extent to which such effects might also occur in other species. The potential for manipulation of variables of interest is key to the value of experimental research, and I have argued that in the context of cultural evolution experiments, two approaches in particular are useful from the perspective of understanding the scope of, and limits on, cumulative culture. The first of these approaches, now well represented in the existing literature, involves manipulation of the conditions under which participants complete a particular task. This allows researchers to determine (within the context of that task) the constraints on, and prerequisites for, effective cumulative improvement over learner generations. This helps to identify key requirements for cumulative culture in studies involving samples with recognized capacities for cumulative culture (i.e., adult humans).

The second approach, which has yet to be exploited to its full potential, involves manipulation of task features themselves. This would allow researchers to identify whether there are circumstances under which cumulative improvement is theoretically possible in studies involving samples (such as nonhuman species) for which cumulative culture has not been convincingly documented in natural populations. This helps to pin down the limiting factors that prevent such cumulative improvements from being observed in practice under more realistic conditions, or in different contexts or domains.
